# Promising Anticancer Activities of *Alismatis rhizome* and Its Triterpenes via p38 and PI3K/Akt/mTOR Signaling Pathways

**DOI:** 10.3390/nu13072455

**Published:** 2021-07-18

**Authors:** Eungyeong Jang, Jang-Hoon Lee

**Affiliations:** 1 Department of Internal Medicine, College of Korean Medicine, Kyung Hee University, Seoul 02447, Korea; obliviona79@naver.com; 2 Department of Internal Medicine, Kyung Hee University Korean Medicine Hospital, Seoul 02447, Korea

**Keywords:** *Alismatis rhizome*, alisol, p38, PI3K, AKT, mTOR, tumor

## Abstract

The flowering plant genus *Alisma*, which belongs to the family Alismataceae, comprises 11 species, including *Alisma orientale*, *Alisma* *canaliculatum*, and *Alisma* *plantago-aquatica*. *Alismatis rhizome* (Ze xie in Chinese, Takusha in Japanese, and Taeksa in Korean, *AR*), the tubers of medicinal plants from *Alisma* species, have long been used to treat inflammatory diseases, hyperlipidemia, diabetes, bacterial infection, edema, oliguria, diarrhea, and dizziness. Recent evidence has demonstrated that its extract showed pharmacological activities to effectively reverse cancer-related molecular targets. In particular, triterpenes naturally isolated from *AR* have been found to exhibit antitumor activity. This study aimed to describe the biological activities and plausible signaling cascades of *AR* and its main compounds in experimental models representing cancer-related physiology and pathology. Available in vitro and in vivo studies revealed that *AR* extract possesses anticancer activity against various cancer cells, and the efficacy might be attributed to the cytotoxic and antimetastatic effects of its alisol compounds, such as alisol A, alisol B, and alisol B 23-acetate. Several beneficial functions of triterpenoids found in *AR* might be due to p38 activation and inhibition of the phosphatidylinositol 3-kinase (PI3K)/protein kinase B (Akt)/mammalian target of rapamycin (mTOR) signaling pathways. Moreover, *AR* and its triterpenes inhibit the proliferation of cancer cells that are resistant to chemotherapy. Thus, *AR* and its triterpenes may play potential roles in tumor attack, as well as a therapeutic remedy alone and in combination with other chemotherapeutic drugs.

## 1. Introduction

Cancer remains a crucial public health issue, with a continuous increase in occurrence and mortality. According to the Global Health Estimates 2020 by the World Health Organization (WHO), cancer ranked as the first or second most common cause of deaths at ages < 70 years in 112 of a total of 183 countries in 2019 [1]. In addition, approximately 10 million cancer deaths were reported in 2020. However, the number of newly diagnosed cases and mortality is predicted to increase continuously in the future [1]. Despite ongoing studies and great advances, such as gene therapy and precision oncology beyond the limitations of previous therapeutic strategies against cancer, many patients have experienced considerable economic burden and unfavorable side effects that cause tumors to quickly grow and metastasize.

Among the numerous mechanisms affecting tumorigenesis, p38 mitogen-activated protein kinase (MAPK) and phosphatidylinositol 3-kinase (PI3K)/protein kinase B (Akt)/mammalian target of the rapamycin (mTOR) pathway are hallmarks of tumors. They are often targeted by various chemotherapeutic agents. Emerging evidence indicates that, at the cellular level, defects in p38 activation and PI3K/Akt/mTOR inhibition strongly facilitate pro-oncogenic functions for cancer survival [2,3]. Hence, these pathways are regarded as attractive targets for developing new anticancer drugs, and several clinical studies using candidates targeting p38 and PI3K/Akt/mTOR are ongoing [4,5]. However, systemic side effects and limited efficacy as a single treatment make these regulators difficult to be approved for clinical application to treat cancer [5,6].

Herbal medicines have been increasingly considered as potential sources of anticancer therapeutic methods because they can enhance the effects and reduce the adverse effects of conventional chemotherapy. Herbal plants themselves are effective enough to target cancer cells without severe toxicity to normal cells [7]. In particular, terpenoids, the largest phytochemical class of herbal plants, exert pharmacological properties to control malignant cells. Several terpenoids, such as artemisinin, tanshinone IIA, oridonin, and lycopene, are candidate bioactive compounds for antitumor drug discovery [8]. *Alismatis rhizome* (*AR*) has been commonly used to ameliorate a wide spectrum of disorders, such as hypertension, diabetes, edema, dizziness, hyperlipidemia, inflammation, oliguria, and leukorrhea [9,10]. Recently, accumulating experimental studies have shown that *AR* and its compounds display significant pharmacological activities against various types and stages of cancers, as well as anti-inflammatory, antihyperlipidemia, diuretic, and neuroprotective effects [9]. In particular, triterpenoids, novel candidates for anticancer drugs, are predominantly found in rhizomes of *Alisma* plants, and several triterpene components isolated from *AR* have been reported to exhibit prominent anticancer activities [11,12]. Among the triterpenoids from *AR*, alisol A (AA), alisol B (AB), alisol B 23-acetate (AB23A), and alisol F 24-acetate (AF24A) might be active compounds with pharmacological effects in cancers, as is shown by the accumulating evidence. Moreover, the underlying mechanisms of these materials appears to be closely associated with the downregulation of p38 and PI3K/Akt/mTOR signaling cascades.

However, no review has extensively demonstrated the linkage between *AR* and cancer, its molecular mechanisms, and the identification of bioactive constituents of *AR* responsible for its efficacy. Therefore, this review summarizes the available findings of the anticancer properties and mechanisms of action of *AR* and its compounds (AA, AB, AB23A, AF24A) using cellular and animal models to present new insights into the pharmacological evidence of these materials against tumors.

## 2. Protostane-Type Triterpenes from *AR*

**Figure 1:**
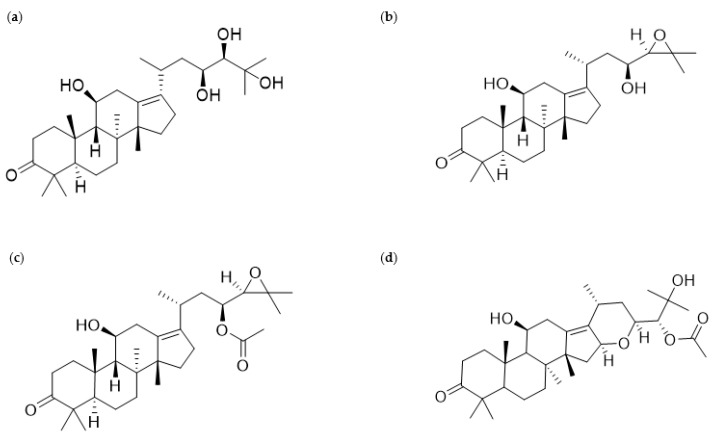
Chemical structures of alisol A (AA), alisol B (AB), alisol B 23-acetate (AB23A), and alisol F 24-acetate (AF24A), which belong to triterpenes isolated from *Alismatis rhizome* (*AR*): (**a**) AA (PubChem a compound identifier (CID): 15558616, C_30_H_50_O_5,_ molecular mass 490.7 g/mol), (**b**) AB (PubChem CID: 15558620, C_30_H_48_O_4,_ molecular mass 472.7 g/mol), (**c**) AB23A (PubChem CID: 14036811, C_32_H_50_O_5,_ molecular mass 514.7 g/mol), (**d**) AF24A (PubChem CID: 76310823, C_32_H_50_O_6,_ molecular mass 530.7 g/mol).

According to previous phytochemical investigations on crude *AR* since the 1960s, more than 200 chemical compounds have been isolated and identified from the plant [9]. The principal bioactive constituents obtained from *AR* can be largely categorized as triterpenes, sesquiterpenes, diterpenes, flavonoid compounds, phenylpropionic acids, and alkaloids [9]. Among these components, protostane-type triterpenes isolated from *AR* have been reported to exert various biological functions, including anti-inflammatory, antibacterial, and antitumor activities [9]. In particular, 13 protostane triterpenoids, including AA (Figure 1a), AB (Figure 1b), alisol F (AF), and AB23A (Figure 1c), among approximately 84 natural protostane-type triterpenes, displayed significant anticancer effects against HepG2 cells [13]. Moreover, triterpenoids from *AR*, such as AA, alisol A 24-acetate, 25-*O*-ethylalisol A, 11-deoxyalisol A, alisol E 24-acetate, alisol G, and AB23A, have been reported to show moderate inhibition in H460, MCF-7, and PC-3 cells, which were influenced by the 3-oxo-protostane framework, the cytotoxic structure–activity relationship [14]. Based on recent investigations on antiproliferative and antimetastatic effects and underlying mechanisms of *AR* against human cancer cells, this review focuses on *AR* and its four protostane triterpenoids AA, AB, AB23A, and AF24A (Figure 1d), all of which have been reported to possess anticancer activities (Figure 1).

## 3. Methodology

A total of four search engines (PUBMED, SCOPUS, Web of Science, and Google Scholar) were used to collect previous studies that were published by March 2021 (Last search date, 31 March 2021). To obtain relevant studies, we used the following key words: (“*Alisma*” OR “Ze xie” OR “Takusha” OR “Taeksa” OR “alisol”) AND (“cancer” OR “tumor”). In addition, reference-based searches were also conducted manually to find as many subject articles as possible. After eliminating duplicated and unrelated articles, a total of 22 studies were collected for the literature review of the anticancer effects of *AR* and its alisol derivatives.

## 4. Anticancer Properties of *AR* and Its Triterpenes

### 4.1. Antiproliferative Effects

The hallmark of tumors is the uncontrolled proliferation of cancer cells, and abnormal cell growth accelerates the development and progression of malignancy. Hence, cytotoxic impacts targeting tumor cells might be a clue in overcoming cancer. Most of the current anticancer agents are projected to suppress the survival and growth of tumor cells. In particular, apoptosis induction and cell cycle arrest might be important molecular pathways that control the initiation and execution of cancer cell overgrowth. Moreover, the autophagic process can suppress tumorigenesis because it is implicated in removing damaged intracellular organelles and proteins [15]. Thus, the therapeutic goals of anticancer drugs have focused on targeting the machinery of apoptotic cell clearance, cell cycle checkpoints, and autophagic recycling processes, since apoptosis deregulation, accelerated cell division rates, and autophagy defects are commonly found in different human tumors.

*AR* has been reported to inhibit the survival of AGS gastric and HeLa cervical tumor cells. The mechanism of antiproliferative activities of *A. canaliculatum* extract (300–500 μg/mL) in AGS is mainly mediated by a series of cellular proteins and enzymes, including Bax, Bcl-2, survivin, poly (ADP-ribose) polymerase (PARP), caspase-3, and -9, which are involved in apoptosis processes [16]. In addition, the proportion of AGS cells in the sub-G1 phase and the depolarization of the mitochondrial membrane potential were significantly increased in the presence of *A. canaliculatum* extract [16]. Moreover*, A. canaliculatum* extract activated p38 MAPK phosphorylation, with no significant changes in extracellular signal-regulated kinase (ERK) and c-JUN N-terminal kinase (JNK), and increased reactive oxygen species (ROS) production in AGS cells. The ROS-stimulated p38 pathway has been reported to play a pivotal role in initiating tumors, including breast cancer [2]. *A. canaliculatum* extract (20 μg/mL) showed no significant cytotoxicity but attenuated the motility of MDA-MB-231 breast cancers exposed to tumor necrosis factor (TNF)-α [17]. This suggests that the efficacy dosage of *A. canaliculatum* might vary depending on the cell types and therapeutic targets, such as tumor growth and metastasis. Similarly, AA showed no cytotoxicity against breast cancers MDA-MB-453 [18], but its treatment resulted in a marked reduction in cellular growth rate, with an average half-maximal inhibitory concentration (IC_50_) value of 8.112 μm in MDA-MB-231 cells by inducing G1 cell cycle arrest and autophagy-dependent apoptosis via p38 activation and oxidative DNA damage induction [19]. Autophagy-dependent apoptosis by AA in MDA-MB-231 cells was also observed in another study which suggested underlying pathways, such as the inactivation of AKT, mTOR, 70S6K, and p65 NF-κB [18]. Its antiproliferative activity against MDA-MB-231 cells was superior to AB, AB23A, and alisol B 24-acetate [19]. However, a methyl thiazolyl tetrazolium (MTT) assay for the quantitation of MCF-7 breast cancer cell growth showed that the inhibitory effect of AA was weaker than that of AB and stronger than that of their 1:1 mixture [20]. Consequently, AA and AB showed differential sensitivities to two types of breast cancer cells, MDA-MB-231 and MCF-7, respectively. Both compounds might be involved in similar mechanisms of enhancing the autophagic process to induce apoptosis and cell cycle arrest, thus leading to suppression of cancer proliferation. In regard to the antiproliferative effects of AB against MCF-7 and MDA-MB-231, AB treatment blocked cell cycle progression from G1 phase in both cell lines [21,22]. The presence of AB increased autophagic vacuoles and LC3-II protein in MCF-7 cells via adenosine monophosphate (AMP)-activated protein kinase (AMPK) activation and p70S6 kinase suppression, and it induced apoptosis in MDA-MB-231 cells via the suppression of AKT, mTOR, p65 NF-κB, and an increase in p38 proteins [21,22]. Among alisol derivatives, AB23A is effective in the most diverse tumor types, including liver, stomach, colon, kidney, lung, ovarian cancers, and leukemia. AB23A significantly suppressed tumor overgrowth in liver, gastric, and prostate cancers [23,24,25,26]. Activated caspase 3 might be crucial for pro-apoptosis when cocultured with AB23A in Hep3B [23], SGC7901 [24], AGS [26], and PC-3 cells [25]. Based on cell cycle analysis using flow cytometry, AB23A hampered G2/M transition in Hep3B cells [23] and induced G0/G1 accumulation in PC-3 cells [25]. In particular, AB23A exerted more powerful anticancer effects on A549 and NCI-H292 lung cancers than AA, alisol A 24-acetate, AF24A, and alisol O via apoptosis induction by increasing ROS generation [27], and AB23A induced G0/G1 arrest and increased apoptotic A549 cells by downregulating the PI3K/Akt/mTOR signaling [28]. However, there was no cytotoxicity induced by AB23A in normal human bronchial epithelial cells 16HBE [27] and normal human lung epithelial cells BEAS-2B [28]. Similarly, AB23A suppressed HepG2 cell growth by inducing G1 arrest and increasing the percentage of apoptotic cells without affecting LO2 normal cells [29]. In addition to in vitro models, AB23A significantly reduced tumor volume and weight by inducing apoptosis in BALB/c mice inoculated with SK-HEP-1 [30] (Table 1).

**Figure 2:**
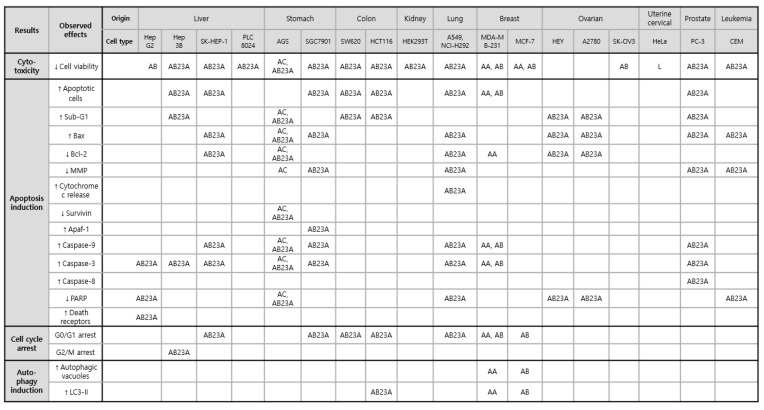
Cytotoxic effects via apoptosis induction, cell cycle arrest, and autophagy induction of *AR* and its triterpenes in various tumors: AC, *A. canaliculatum*; L, lectin obtained from *A. orientale*; AA, alisol A; AB, alisol B; AB23A, alisol B 23-acetate; Bax, Bcl2-associated X protein; Bcl, B-cell lymphoma; MMP, mitochondrial membrane potential; Apaf-1, apoptotic protease activating factor 1; PARP, poly ADP ribose polymerase. Upward pointing arrows (↑) and downward pointing arrows (↓) indicate an increase or a decrease in a numerical value, rate, change, or degree, respectively.

These observations indicate that AR, AA, AB, and AB23A might stimulate various signs of pro-apoptotic signaling, cell cycle arrest, and autophagic degradation, which impede the overgrowth of many cancer cells (Figure 2). In particular, AB showed strong inhibition in HepG2 cells, with an IC_50_ value of 32.57 μmol/L, which was lower than those of kaempferol and quercetin (>200 μmol/L). The antiproliferative effect of AB can be influenced by the oxidation at C-16 and the C-13 and C-17 double bond [31]. These key mechanisms behind such antiproliferative activity might be slightly different from the mechanism of action of existing chemotherapeutic agents that can induce acquired resistance or unfavorable side effects. For example, AB23A attacked liver and lung cancer cells without inducing cytotoxicity to normal hepatic LO2, bronchial 16HBE, and epithelial BEAS-2B cells. In addition, AB23A increased G2/M arrest through the cell division control protein 2 homolog (CDC2) kinase-independent pathway, whereas taxol-induced G2/M arrest was stimulated by CDC2 activation [32]. Hence, detailed molecular mechanisms underlying the antiproliferative activities of AR, AA, AB and AB23A should be further investigated to use these materials in the treatment of various cancers.

### 4.2. Antimigratory and Anti-Invasive Effefcts

The migratory and invasive ability of tumor cells initiates and accelerates a cascade of multistep processes that promote tumor malignancy. Metastasis is responsible for approximately 90% of cancer-related mortality, and patients with metastatic cancer generally have a poor prognosis due to failure of cancer therapy [39]. Regulating the dynamics of sequential processes and identifying molecular targets relevant to invasion and metastasis will help manage cancer growth and malignancy. In particular, matrix metalloproteinases (MMPs) and different chemokines participate in metastatic cascades, such as dissemination of cancer cells, intravasation, extravasation, etc. [40]. Hence, targeting the enzymatic activity of MMPs and chemokines release might play a crucial role in decreasing cancer aggressiveness and invasive potential, and these markers might be useful for evaluating prognosis and therapeutic efficacy.

The ethanol extract of *A. canaliculatum* suppressed MDA-MB-231 cell migration and invasion [17], and *A. orientalis* showed positive inhibitory effects on spontaneous metastasis in C57BL/6 mice transplanted with Lewis lung carcinoma [41]. In particular, the attenuation of TNFα-induced C-X-C motif chemokine receptor 3 (CXCR3) and CXCR10 mRNA was strongly correlated with the inhibitory effects of *A. canaliculatum* extract on MDA-MB-231 cell motility and migration without toxicity [17]. Moreover, the downregulation of CXCR3 and CXCR10 expression was mainly dependent on the blockade of the nuclear translocation of IκB kinase (IKK)-mediated nuclear factor kappa light chain enhancer of activated B cells (NFκB) [17]. As expected, the suppression of *p*-IKKα/β, *p*-IκB, and *p*-p65 proteins by *A. canaliculatum* was observed [17], and the extract was possibly involved in the regulation of epithelial-to-mesenchymal transition genes, one of key mechanisms of tumor metastasis [42]. AA was shown to lower MDA-MB-231 cell migration and invasion rates using a wound healing assay and a transwell assay by decreasing MMP-2 and MMP-9 protein levels [18]. AKT/mTOR and p65 NFκB signaling pathways are likely to be considered key elements in mediating the underlying mechanisms of antimetastatic activities of AA. Based on these experimental results, we suggest that *A. canaliculatum* and AA could significantly suppress metastatic responses by targeting pro-metastatic chemokines and MMP expression in MDA-MB-231 cells with higher invasive potential. AB23A possesses antimetastatic effects in human non-small cell lung cancer A549, human hepatoma SK-HEP-1, and human ovarian cancer HEY cells. In particular, AB23A significantly decreased SK-HEP-1 and HEY cell migration and invasion by inhibiting MMP-2 and MMP-9 protein and gene expression [30,33]. Similar to AA, MMP downregulation by AB23A is mediated by the blockade of PI3K/AKT phosphorylation [30] (Table 2).

In summary, *A. canaliculatum*, *A. orientalis*, AA, and AB23A might regulate MMP activity and chemokine production in highly invasive cancer cells, such as SK-HEP-1, HEY, and MDA-MB-231, by hindering molecular downstream pathways, such as PI3K/AKT/mTOR and NFκB which activations trigger metastatic progression. This indicated that these materials could be involved in altering a series of metastatic events, helping tumor cells achieve invasive ability.

### 4.3. Antiresistant Effects

Chemotherapeutic agents play a crucial role in either direct or indirect inhibition of tumor growth, and they have shown significant therapeutic efficacy for different types of cancer. However, multidrug resistance (MDR) is a major obstacle that limits complete remission by chemotherapy [43]. MDR is a phenomenon in which tumor cells do not respond to anticancer drugs, despite the administration of a concentration capable of killing cells. It is a survival strategy for malignant cells during prolonged chemotherapy. In general, the development of MDR displays simultaneous resistance to various drugs, with chemical structures and mechanisms of action that are completely different from chemotherapeutic agents that show resistance [44].

Several molecular mechanisms have been suggested to contribute to MDR. Among them, the MDR-1 gene encoding human *p*-glycoprotein (Pgp), a transmembrane protein that actively pumps intracellular anticancer drugs, is known to develop MDR [45]. The elevated overexpression of Pgp in drug-resistant tumor cells is commonly regarded as a classic molecular mechanism of MDR. Hence, reversing MDR by inactivating the function of drug efflux pumps and increasing the sensitivity of cancer cells to chemotherapy can be a solution to overcome tumor recurrence and progression, particularly in tumors with a high frequency of drug resistance, such as non-small cell lung cancer (NSCLC) [46], ovarian adenocarcinomas [47], and acute lymphoblastic leukemia [48].

*AR* and its alisol derivatives, including AB23A and AF24A, significantly inhibited tumor cell survival in four acquired resistant cancer cell lines. When studying *AR* inhibited tumor growth rate in HepG2 cells resistant to vinblastine, actinomycin D, puromycin, paclitaxel, and doxorubicin, the addition of 25 μg/mL of its 95% ethanol extract significantly lowered the resistance factor of the IC_50_ ratio of HepG2 MDR subline to HepG2, thus increasing drug sensitivity approximately 3 to 150 times higher than that in HepG2 cells [49]. The effect of 25 μg/mL *AR* enhancing sensitivity to anticancer drugs in HepG2 MDR subline was more potent than that of 10 μm verapamil [49]. The significant cytotoxicity induced by *AR* extract in drug-resistant HepG2 cells mainly contributed to G2/M arrest [49]. Similarly, concomitant treatment with AB23A restored MDR by increasing the percentage of resistant HepG2 cells in the G2/M phase, as demonstrated by cell cycle analysis using flow cytometry [50]. Moreover, AB23A displayed a more potent reversal effect than the Pgp inhibitor verapamil in HepG2 cells resistant to vinblastine, although its activity was weaker than verapamil in resistant K562 leukemia cells [50]. Furthermore, AB23A and AF24A improved anticancer efficacy through apoptosis induction in tumors that frequently develop recurrence, similar to A2780 ovarian cells resistant to paclitaxel and MCF-7 breast cancer cells resistant to digoxin, respectively [33,51]. Both compounds showed a significant cytotoxicity in resistant cells, while the inhibitory effects of paclitaxel and verapamil were diminished due to high resistance [33,51].

The cytotoxicity of *AR*, AB23A, and AF24A through apoptosis induction or cell cycle arrest in drug-resistant cells was mostly accompanied by the increased accumulation of chemotherapeutic agents in MDR tumor cells [49,50,51]. They significantly elevated intracellular concentrations and increased the migration of anticancer drugs, such as doxorubicin. AB23A and AF24A might act as chemosensitizers because they exert synergistic effects by showing a combination index of less than 0.8, combined with conventional chemotherapy in HepG2- and MCF-7-resistant cells [50,51]. Moreover, AF24A was more potent in decreasing the efflux ratio of digoxin in Caco-2 colorectal adenocarcinoma overexpressing Pgp than verapamil, a powerful Pgp inhibitor [51] (Table 3).

**Figure 3:**
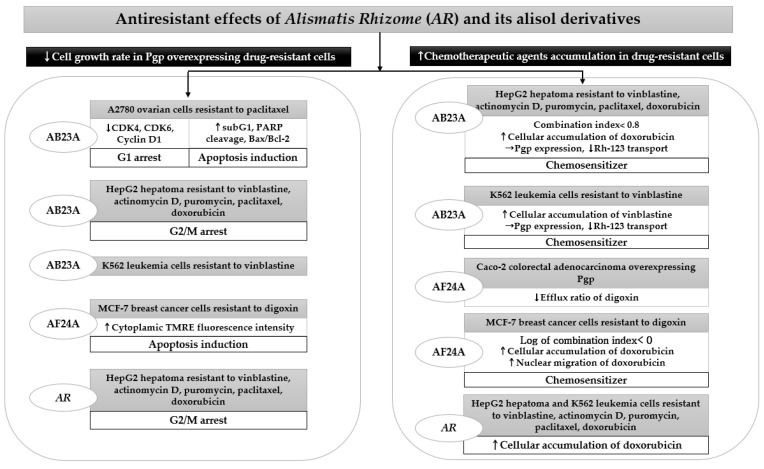
Antiresistant effects of *AR* and its triterpenes in drug-resistant cancer cells: AB23A, alisol B 23-acetate; AF24A, alisol F 24-acetate; *AR*, *Alismatis rhizome*; CDK, cyclin-dependent kinase; Bax: Bcl2-associated X protein; Bcl: B-cell lymphoma; Rh-123, rhodamine 123; Pgp, *p*-glycoprotein. Upward pointing arrows (↑) and downward pointing arrows (↓) indicate an increase or a decrease in a numerical value, rate, change, or degree, respectively.

In summary, the addition of *AR*, AB23A, and AF24A could help sensitize chemoresistant cancer cells to conventional anticancer drugs, thus eventually preventing chemotherapy failure or tumor recurrence induced by a decreased sensitivity of tumor cells. Using ADME/Tox software, AF24A was found to show a higher probability as a Pgp inhibitor than AB23A (Figure 3). Hence, further studies are needed to confirm the development potential of *AR* and its chemical constituents as MDR reversal agents.

## 5. The p38 and the Phosphoinositide 3-Kinase-AKT-Mammalian Target of Rapamycin (PI3K/AKT/mTOR) Pathway

**Figure 4:**
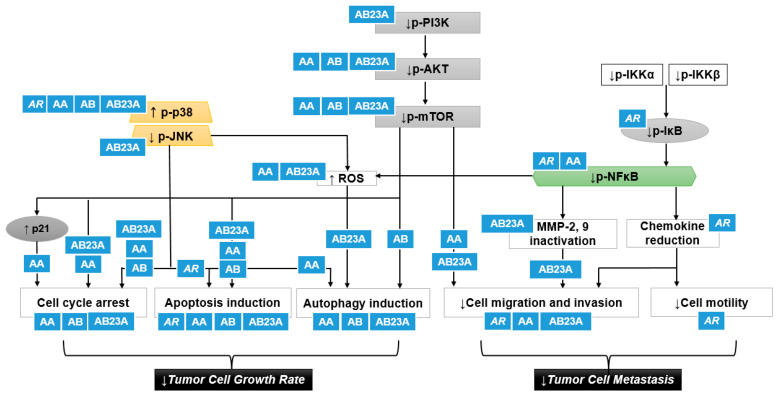
A putative scheme for the p38 activation and PI3K/Akt/mTOR inhibition by *AR* and its triterpenes in suppressing cancer cell proliferation and tumor cell metastasis: *AR*, *Alismatis rhizome*: AA, alisol A; AB, alisol B; AB23A, alisol B 23-acetate; JNK, c-Jun N-terminal kinase; ROS, reactive oxygen species; IKK, IκB kinase. Upward pointing arrows (↑) and downward pointing arrows (↓) indicate an increase or a decrease in an expression or production level, rate or degree, respectively.

To explore the molecular mechanisms responsible for the anticancer activities of *AR* and its triterpenes (AA, AB, and AB23A), we thoroughly reviewed the signaling pathways that regulate apoptosis, cell cycle arrest, autophagy, migration, or invasion in cellular models treated with these materials. As illustrated in Figure 4, the p38 MAPK and PI3K/AKT/mTOR pathways have been identified as key molecular cascades underlying the antiproliferative and antimetastatic effects of *AR* and its alisol derivatives in various malignant cells. p38 MAPK is frequently highly suppressed in cancer cells, and this pathway has been implicated in different stages of tumor cell overgrowth and metastasis [52]. In addition, p38 MAPK activation plays a critical role in regulating tumorigenesis and metastasis through its interaction with different signaling pathways, such as PI3K/Akt/mTOR [53,54]. The activated PI3K/Akt/mTOR pathway is also considered a typical feature of cancer [3]. Hence, p38 and PI3K/Akt/mTOR can be key therapeutic targets for cancer treatment.

For the activation of p38 MAPK, the phosphorylation of p38 increased by *A. canaliculatum* extract and AB23A resulted in the suppression of cellular proliferation of AGS gastric cancer cells by inducing sub-G1 peak, regulating Bax and Bcl-2 proteins, inhibiting the expression of survivin protein, and increasing PARP cleavage, indicating cell apoptosis [16,26]. AB23A might play a crucial role in the phosphorylation of p38, contributing to the antiproliferative effects of *A. canaliculatum* extract in AGS cells. In addition, AA and AB showed strong cytotoxic effects by increasing p38 expression in MDA-MB-231 breast cancer cells, with IC_50_ values of 8.112 and 13.96 μm, respectively [19,22]. The p38 activation induced by AA and AB in an aggressive MDA-MB-231 cell line induced LC3-II accumulation and increased caspase activity, respectively [19,22]. These p38-activated cytotoxic autophagy and apoptosis induction may be underlying mechanisms for the antiproliferative effects of AA and AB for the treatment of breast cancers with high metastatic potential.

The phosphorylation of Akt has been implicated in regulating cell proliferation, apoptosis, autophagy, and cell motility in cancer, and *p*-Akt overexpression is one of the major therapeutic targets in treating malignant tumors [55]. Akt is considered a downstream effector of PI3K, and the inhibition of mTOR is closely implicated in the PI3K/Akt pathway [3]. The antiproliferative effects of AA and AB in MDA-MB-231 cells depend on the suppression of Akt signaling, as well as p38 activation [19,22]. The interference of AA and AB in the Akt signaling accelerating cell growth and metastasis may influence the antimetastatic effects of *A. canaliculatum* via the suppression of NFκB-related protein production in MDA-MB-231 cells [17] because NFκB activation occurs via Akt phosphorylation [56]. The downregulation of the Akt/mTOR pathway was also involved in the inhibition of cell migration and invasion by the treatment of AA in MDA-MB-231 [18]. AB23A induced mitochondrial apoptosis through the regulation of PI3K/Akt pathway in A549 [28], SK-HEP-1 [30], and SGC7901 [24] cells, which resulted in overgrowth inhibition of cancer.

Interestingly, the treatment of MDA-MB-231 cells with AA and AB was associated with a significant cytotoxicity via the activation of p38 and the blockade of Akt/mTOR and NFκB signaling simultaneously [18,22]. Among the mechanisms frequently observed after the treatment of *AR* and its triterpenes in human cancer cells, the PI3K/Akt/mTOR pathway is likely to be the most frequently interrupted pathway [18,21,22,24,28,29,30]. In particular, the antiproliferative and antimetastatic activities of AB23A in SK-HEP-1 cells via the PI3K/Akt pathway [30] and AA in MDA-MB-231 cells via the Akt/mTOR pathway [18] can offer clinical applicability for the treatment of tumors and the prevention of metastasis. Hence, further studies are required to identify specific mechanisms of *AR* and its triterpenes in various types of human cancer cells, which could present comprehensive evidence regarding efficacy and active compounds.

## 6. Discussion

The pharmacological effects of *AR* and its triterpenes in suppressing tumor proliferation and metastasis in various human cancer cells have gained attention because of their potential as anticancer therapies. This review indicates that *AR* and its triterpene can inhibit the proliferation of various cancers by inducing cell cycle arrest, apoptosis, and autophagy, as suggested in Table 1 and Figure 2. Most studies regarding the cytotoxic effects of *AR* and its triterpene have shown that the inhibition of cell viability, on which the growth of cancer depends, is a useful target for the suppression of cell proliferation.

The presence of *AR*, AA, AB, and AB23A resulted in a significant decrease in cell viability and lowered cell growth rate using MTT assay in various malignant cells, such as AGS [16,26,38], SGC7901 [24], SW620 [38], HCT116 [38], HEK293T [38], MDA-MB-231 [18,19,22], MCF-7 [20,21], HEY [33], A2780 [33], SK-OV3 [35], HeLa [36], PC-3 [25], CEM [37], HepG2 [29,34], Hep3B [23], SK-HEP-1 [30], A549 [27,28], and NCI-H292 [27]. The intrinsic mitochondrial pathway is prevalently involved in the antiproliferative effects of *AR*, AA, AB, and AB23A. The sub-G1 peak, mitochondrial membrane depolarization, and the regulation of pro-apoptotic and anti-apoptotic proteins induced by the materials led to caspase effector activation, thus eventually increasing apoptotic cancer cells. Interestingly, AB23A increased caspase-3 cleavage in HepG2 cells through death receptors, with no toxicity in normal LO2 liver cells, which stimulates the extrinsic apoptotic pathway [29]. Specifically targeting death receptors in cancer cells can be attractive for triggering cancer death [57]. In addition, deactivation in both intrinsic and extrinsic apoptosis pathways promotes tumor malignancy and drug resistance [57]. Thus, AB23A can serve as a novel compound to repress cancer cells by targeting both intrinsic and extrinsic apoptosis pathways. In regard to cell cycle arrest, the treatment of AA and AB induced G0/G1 cell cycle arrest in breast cancer cells such as MDA-MB-231 and MCF-7 that allow cancer cells the opportunity to repair their own damaged DNA and to hinder uncontrolled cell division [18,19,21,22]. AB23A was shown to control cell cycle transition of G2 to M phase in Hep3B [23], as well as G0/G1 arrest in SK-HEP-1 [30], SGC7901 [24], SW620 [38], HCT116 [38], and A549 [28], which might support the anticancer effects of the compound. In addition, AA, AB, and AB23A exerted their antiproliferative activities through the increase in the formation of autophagy vacuoles and the expression of LC3-II protein within tumor cells [18,19,21,38], thus eventually degrading cytoplasmic organelles.

As suggested in Table 2, alteration in MMPs’ production and chemokine activity induced by *AR*, AA, and AB23A has been implicated in suppressing the development of cell migration and invasion. Importantly, AA and AB23A exerted a significant inhibition in cell migration and invasion, the properties of which were confirmed by a wound healing assay and a transwell assay in SK-HEP-1 liver adenocarcinoma, metastatic ovarian cancer HEY cells, and MDA-MB-231 breast adenocarcinoma with greater metastatic capacity [18,30,33], respectively. In addition*, A. orientale* extract repressed spontaneous metastasis of highly invasive Lewis lung carcinoma in C57BL/6 [41]. Since metastasis is a major event contributing to the mortality of cancer patients, further studies are required to identify active compounds of *AR* and the functional pathways that are especially efficacious for the prevention and treatment of tumor metastasis.

As previously mentioned, *AR*, AA, AB, and AB23A have been found to show anticancer functions against various tumors by modulating the p38 and PI3K/Akt/mTOR pathways. Activation of p38 and inhibition of PI3K/Akt/mTOR induced by these materials in cancer cells can induce pro-apoptotic changes, cell cycle arrest, the autophagic process, and downregulate cell migration, resulting in both cellular proliferation suppression and metastasis inhibition. Additionally, p38 inhibition and PI3K/Akt/mTOR activation in cancer cells frequently cause drug resistance [6,58]. This suggests that *AR* and its compounds that mediate these pathways in tumors can improve chemotherapy resistance. Notably, MDR improvement was observed using *AR*, AB23A, and AF24A in chemoresistant hepatoma, leukemia, breast cancer, ovarian cancer, and colorectal adenocarcinoma.

Among various tumor types, it has mainly been noted that the treatment of *AR* and its triterpenes resulted in significant suppression of liver, breast, and gastric cancer cells, particularly cell viability and migration. First, the antiproliferative effects of AB and AB23A appear to be promising in liver cancer. Hepatocellular carcinoma (HCC) is the most prevalent liver cancer and one of the major causes of cancer-related deaths worldwide [59]. Against the highly aggressive HCC cell lines, HepG2, Hep3B, and SK-HEP-1, showed significant inhibition of cell growth rate via cell cycle arrest and apoptosis induction. In HepG2 cells, IC_50_ values of AB and AB23A were 32.57 μm and 17.82 μm, respectively. AB showed the most effective activity on inhibiting HepG2 viability among nine major compounds (quercetin, kaempferol, hederagenin, β-sitosterol, stigmasterol, formononetin, isorhamnetin, licochalcone a, and AB) of Bushen-Jianpi, the decoction of which is frequently used to treat liver cirrhosis and cancer [34]. AB23A lowered migration and invasion rates in SK-HEP-1 cells and played roles as chemotherapy and chemosensitizers in HepG2 cells resistant to vinblastine, actinomycin D, puromycin, paclitaxel, and doxorubicin. Recently, fatty liver has been considered an emerging etiological cause contributing to HCC development [60]. *A. orientale* has been shown to exert bioactive effects against nonalcoholic fatty liver disease [61]. Hence, *AR* and its triterpene might be promising for the treatment of HCC. Second, *AR*, AA, AB, and AF24A regulated some cancer-related markers in human breast cancer cells, MDA-MB-231, and MCR-7 cells. The inhibitory effects of AB on cellular overgrowth were better than those of AA in MCF-7 cells. However, AA markedly inhibited cell division via cell cycle arrest, apoptosis, and autophagy induction and migration in MDA-MB-231 cells. Both the downregulation of AKT/mTOR and activation of p38 can be implicated in the regulation of overgrowth, migration, and invasion induced by AA treatment in MDA-MB-231 cells. Third, *AC* species belonging to *Alisma* genus are particularly excellent at inhibiting the cell viability of AGS gastric cancer cells via the mitochondria-dependent pathway [16]. Similarly, AB23A reduced AGS cell proliferation by increasing sub-G1 proportion and regulating Bcl-2, BAX, and caspase expression [26]. In addition, both *AC* extract and AB23A activated p38 kinase in AGS cells [16,26]. Thus, it appears that AB23A is a crucial compound contributing to the antiproliferative effects of *AC* in AGS cells.

In summary, *AR* and its triterpenes exhibit significant cytotoxic activity against cancer cells. Significant alterations in p38 and PI3K/Akt/mTOR after *AR*, AA, AB, and AB23A treatments in various cancer cells were observed. In addition, these materials can be used as adjuvants in combination therapy with conventional anticancer therapy during prolonged chemotherapy because of their chemosensitizing properties. However, most of the experiments conducted on *AR* and cancer have been conducted in vitro. Thus, further in vivo studies and phytochemical standardization analyses are needed to identify key compounds to assess the usefulness of *AR* in cancer treatment.

## 7. Conclusions

In conclusion, the present review indicates that *AR* and its triterpenes, including AA, AB, and AB23A*,* possess potent antitumor activities by suppressing cell proliferation, migration, and invasion, mainly by targeting the p38 and PI3K/Akt/mTOR pathways, which account for key molecular mechanisms underlying the pharmacological actions of these materials. In addition, *AR*, AB23A, and AF24A reversed MDR when added to resistant cancer cells. However, since most of experimental results that this review summarized are based on cell models, further cell and animal studies with positive controls and well-designed clinical trials of *AR* and its triterpenes need to be conducted. In addition, further toxicological and pharmacokinetic studies of *AR* and its triterpenes are required to assess appropriate oral dosage to prevent side effects that can occur along with their intake, as well as to improve their efficacy. More in-depth investigations on these aspects will help apprehend the efficacy and targets of action and provide more comprehensive information regarding *AR* and its triterpenes in relation to cancer.

## Figures and Tables

**Table 1:** Antiproliferative effects and molecular mechanisms of *AR* and its triterpenes.

Sources	Country	Models	Doses	Results and Mechanisms	Ref.
*A. canaliculatum* extract	Korea	In vitro, human gastric cancer cells AGS	300–500 μg/mL	**↓** **Cell Growth Rate**	[16]
				**Apoptosis induction** ↑Sub-G1 ↑Mitochondrial membrane depolarization ↑Bax protein ↓Bcl-2 protein ↓Survivin protein ↑PARP cleavage	
				**↑*p*-p38 protein**	
AB23A	Korea	In vitro, human gastric cancer cells AGS	50 μm	**↓** **Cell growth rate**	[26]
				**Apoptosis induction** ↑Sub-G1 ↑Bax protein ↓Bcl-2 protein ↓Survivin protein ↑PARP cleavage ↑Caspase-3,-9 protein cleavage	
				**↑*p*-p38, *p*-ERK, *p*-JNK protein**	
				**ROS generation** ↑Intracellular ROS levels	
AB23A obtained from National Institutes for Food and Drug Control (Beijing, China)	China	In vitro, human ovarian cancer cells HEY	2.5–20 μm	**↓Cell growth rate** (IC_50_: 10.73 μm)	[33]
				**Cell cycle arrest** ↑G1 arrest ↓Cyclin D1, CDK4, CDK6 protein	
				**Apoptosis induction** ↑Sub-G1 ↑PARP cleavage ↑Bax/Bcl-2	
AB23A obtained from National Institutes for Food and Drug Control (Beijing, China)	China	In vitro, human ovarian cancer cells A2780	9–18 μm	**↓Cell growth rate** (IC_50_: 11.21 μm)	[33]
				**Cell cycle arrest** ↑G1 arrest ↓Cyclin D1, CDK4, CDK6 protein	
				**Apoptosis induction** ↑Sub-G1 ↑PARP cleavage ↑Bax/Bcl-2	
AB23A	China	In vitro, human hepatoma HepG2	10, 15, 20 μm	**↓Cell growth rate** (IC_50_: 17.82 F)	[29]
				**Cell cycle arrest** ↑G1 arrest ↓Cyclin D1, CDK4, Rb, *p*-Rb protein	
				**Apoptosis induction** ↑Cells displaying nuclear condensation and fragmentation ↑The percentage of apoptotic cells ↑PARP cleavage ↑Caspase-3 cleavage Through DR3 and DR4/5 death receptors	
				**↓** ***p*** **-mTOR**	
AB purchased from the Standardization Research Center of TCM (Shanghai, China)	Shanghai, China	In vitro, human hepatoma HepG2	6.25–200 μmol/L	**↓Cell growth rate** (IC_50_: 32.57 μm)	[34]
AB23A from *A. plantago-aquatica*	Kyoto, Japan	In vitro, human hepatoma containing hepatitis B virus Hep3B	50 μm	**↓Cell growth rate** (IC_50_: 42.4 μm)	[23]
				Cell cycle arrest ↑G2/M arrest	
				**Apoptosis induction** ↑The percentage of apoptotic cells↑DNA fragmentation ↑Sub-G1 ↑Caspase-3 protein	
AB23A	Shanghai, China	In vitro, human non-small cell lung cancer cells A549	6, 9 mM	**↓** **Cell growth rate**	[28]
				**Cell cycle arrest** ↑G0/G1 arrest	
				**Apoptosis induction** ↑The percentage of apoptotic cells↑Bax protein ↓Bcl-2 protein	
				**↓** ***p*** **-PI3K/AKT/mTOR protein**	
AB23A isolated from the 90% ethanol extract of *A.* *orientale*	Shandong, China	In vitro, human lung cancer cells A549 (adenocarcinoma) and NCI-H292 (mucoepidermoid cancer)	10, 20 μm	**↓** **Cell growth rate**	[27]
				**Apoptosis induction** ↑The percentage of apoptotic cells ↑Bax, Caspase-3,-9 protein ↓Bcl-2, Bcl-xL protein ↑PARP cleavage ↑Mitochondrial membrane depolarization ↑Cytochrome c release	
				**ROS generation** ↑Intracellular ROS levels	
AB23A	Beijing, China	In vitro, human Hepatoma SK-HEP-1	30 μmol/L	**↓** **Cell growth rate**	[30]
				**Cell cycle arrest** ↑G0/G1 arrest	
				**Apoptosis induction** ↑The percentage of apoptotic cells ↑Bax, Caspase-3,-9 protein and mRNA ↓Bcl-2 protein and mRNA	
				**↓*p*-PI3K/AKT protein and mRNA**	
AB23A purchased from Wako Pure Chemical Industries (Osaka, Japan)	Osaka, Japan	In vitro, human gastric cancer cells SGC7901	30 μmol/L	**↓** **Cell growth rate**	[24]
				**Cell cycle arrest** ↑G0/G1 arrest	
				**Apoptosis induction** ↑The percentage of apoptotic cells ↑Bax, Caspase-3,-9 protein ↑Apaf-1 protein ↑Mitochondrial membrane depolarization	
				**↓** ***p*** **-PI3K/AKT protein**	
AB23A	Beijing, China	In vivo, BALB/c mice inoculated with SK-HEP-1	30 μmol/L	**↓Tumor volume and weight**	[30]
				**Apoptosis induction** ↑Bax, Caspase-3,-9 protein and mRNA in tumor xenograft ↓Bcl-2 protein and mRNA in tumor xenograft	
AB	Daejeon, Korea	In vitro, human ovarian cancer cells SK-OV3		**↓Cell growth rate** (IC_50_: 7.5 μg/mL)	[35]
Lectin from *A. orientale*	Jianou, China	In vitro, human uterine cervical cancer cells HeLa	5, 10, 20 μm	**↓Cell growth rate** (IC_50_: 7.3 μm)	[36]
AA purchased from MedChemExpress (Monmouth Junction, NJ, USA)	Hangzhou, China	In vitro, human breast cancer cells MDA-MB-231	20, 40 μm	**↓** **Cell growth rate**	[18]
				**Cell cycle arrest** ↑G0/G1 arrest ↓Cyclin D1 ↑p21	
				**Apoptosis induction** ↑The percentage of apoptotic cells	
				**Autophagy induction** ↑Autophagic vacuoles ↑LC3-II protein	
				**↓** ***p*** **-AKT protein** **↓** ***p*** **-mTOR protein** **↓** ***p*** **-70S6K protein** **↓** ***p*** **-NFκB protein**	
AA	Chengdu, China	In vitro, human breast cancer cells MCF-7	30, 100 μm	**↓** **Cell growth rate**	[20]
AA	Beijing, China	In vitro, human breast cancer cells MDA-MB-231	5, 10, 20 μm	**↓Cell growth rate** (IC_50_: 8.112 μm)	[19]
				**Cell cycle arrest** ↑G1 arrest ↓Cyclin A/D1	
				**Apoptosis induction** ↑The percentage of apoptotic cells ↑Caspase-3,-9 protein ↓Bcl-2 protein	
				**Autophagy induction** ↑LC3-II protein	
				**↑*p*-p38 protein** **↑** **ROS positive cells** **↑** **DNA damage markers**	
AB	Chengdu, China	In vitro, human breast cancer cells MCF-7	10, 30, 100 μm	**↓** **Cell growth rate**	[20]
AA-AB (1:1)	Chengdu, China	In vitro, human breast cancer cells MCF-7	100 μm	**↓** **Cell growth rate**	[20]
AB purchased from Wako Pure Chemical Industries (Osaka, Japan)	Osaka, Japan	In vitro, human breast cancer cells MCF-7	30 μmol/L	**↓Cell growth rate** (IC_50_: 29.9 μmol/L)	[21]
				**Cell cycle arrest** ↑G1 arrest ↑p27	
				**Autophagy induction** ↑Autophagic vacuoles ↑LC3-II protein	
				**↑*p*-AMPK** **↓** ***p*** **-70S6**	
AB purchased from Sigma Chemical Co. (St. Louis, MO, USA)	Zhenjiang, China	In vitro, human breast cancer cells MDA-MB-231	10, 20 μm	**↓Cell growth rate** (IC_50_: 13.96 μm)	[22]
				**Cell cycle arrest** ↑G0/G1 arrest	
				**Apoptosis induction** ↑The percentage of apoptotic cells ↑Caspase-3,-9 protein	
				**↓** ***p*** **-AKT/mTOR protein** **↓** ***p*** **-p65 protein** **↑*p*-p38 protein**	
AB23A purchased from Wako Pure Chemical Industries (Osaka, Japan)	Osaka, Japan	In vitro, human prostate cancer PC-3 cells	30 μm	**↓Cell growth rate** (IC_50_: 13.5 μm)	[25]
				**Apoptosis induction** ↑The percentage of apoptotic cells ↑Mitochondrial membrane depolarization ↑Bax protein ↑Cleaved caspase-3,-9 protein ↑Cleaved caspase-8 protein	
AB23 A from *A. plantago-aquatica* purchased from Nacalai Tesque (Kyoto, Japan)	Taipei, Taiwan	In vitro, human acute lymphoblastic leukemia CEM cells	10^−6^–10^−4^ M	**↓Cell growth rate** (IC_50_: 10^−4^ M)	[37]
				**Apoptosis induction** ↑Mitochondrial membrane depolarization ↑c-myc and Bax mRNA and proteins	
AB23A	China	In vitro, human colon cancer cells SW620 and HCT116	5, 10, 20 μm	**↓Cell growth rate** (IC_50_: 20 μm)	[38]
				**Cell cycle arrest** ↑G1 arrest	
				**Apoptosis induction** ↑The percentage of apoptotic cells ↑Sub-G1 ↑PARP cleavage	
				**Autophagy induction in HCT116** ↑LC3-II protein ↓p62 protein (substrate)	
				**ROS generation** **↑Intracellular ROS levels** **↓** ***p*** **-JNK protein**	
AB23A	China	In vitro, human kidney, gastric, and liver cancer cells HEK293T, AGS, PLC8024	20 μm	**↓** **Cell growth rate**	[38]

AA, alisol A; AB, alisol B; AB23A, alisol B 23-acetate; Bax, Bcl2-associated X protein; Bcl, B-cell lymphoma; PARP, poly (ADP-ribose) polymerase; CDK, cyclin-dependent kinase; Akt, protein kinase B; ERK, extracellular signal-regulated kinase; ROS, reactive oxygen species; JNK, c-Jun N-terminal kinase. Upward pointing arrows (↑) and downward pointing arrows (↓) indicate an increase or a decrease in a numerical value, rate, change, or degree, respectively.

**Table 2:** Antimetastatic effects and molecular mechanisms of *AR* and its terpenes.

Sources	Country	Models	Doses	Results and Mechanisms	Ref.
AB23A	Shanghai, China	In vitro, human non-small cell lung cancer cells A549	6, 9 mM	**↓** **Cell migration and invasion**	[28]
AB23A	China	In vitro, human hepatoma cells SK-HEP-1	30 μmol/L	**↓Cell migration and invasion** ↓Migration and invasion rate using wound healing assay and transwell assay	[30]
				**↓** **MMP-2, 9 protein and mRNA** **↓** ***p*** **-PI3K/AKT protein and mRNA**	
AB23A	Beijing, China	In vitro, human ovarian cancer cells HEY	5, 10 μm	**↓Cell migration and invasion** ↓Migration and invasion rate using wound healing assay and transwell assay	[33]
				**↓** **MMP-2, 9 protein**	
AA Purchased from MedChem Express (Monmouth Junction, NJ, USA)	Hanzhou, China	In vitro, human breast cancer cells MDA-MB-231	5 μm	**↓Cell migration and invasion** ↓Migration and invasion rate using wound healing assay and transwell assay	[18]
				**↓** **MMP-2, 9 protein** **↓** ***p*** **-AKT protein** **↓** ***p*** **-mTOR protein** **↓** ***p*** **-70S6K protein** **↓** ***p*** **-NFκB protein**	
*A. canaliculatum* Purchased from the Kyungdong traditional medicine market (Seoul, Korea)	Seoul, Korea	In vitro, human breast cancer cells MDA-MB-231	20 μg/mL	**↓Cell migration and invasion** ↓TNFα-induced migration rate using wound healing assay ↓TNFα-induced motility causing morphological changes to spindle-like cells	[17]
				**↓** **TNFα-induced CXCR3 and CXCL10 mRNA** **↓** ***p*** **-IKKα/β protein** **↓** ***p*** **-IκB, *p*-p65/RelA protein**	
*A. orientalis*	China	In vivo, C57BL/6 transplanted with Lewis lung carcinoma	10, 20 g/kg/d	**↓** **Spontaneous metastasis**	[41]

AB23A, alisol B 23-acetate; AA, alisol acetate; MMP, matrix metalloproteinases; CXCR3, CXC motif chemokine receptor 3; CXCL10, CXC motif chemokine ligand 10; IKK, IκB kinase. Upward pointing arrows (↑) and downward pointing arrows (↓) indicate an increase or a decrease in a numerical value, rate, change, or degree, respectively.

**Table 3:** Antiresistant effects and molecular mechanisms of AR and its triterpenes.

Sources	Country	Models	Doses	Positive control	Results and Mechanisms	Ref.
AB23A	Beijing, China	In vitro, human ovarian cancer A2780 cells resistant to paclitaxel drug	12–18 μm	paclitaxel 10 μm	**↓Cell growth rate** (IC_50_: 15.18 μm)	[33]
					**Cell cycle arrest** ↑G1 arrest ↓Cyclin D1, CDK4, CDK6 protein	
					**Apoptosis induction** ↑Sub-G1 ↑PARP cleavage ↑Bax/Bcl-2	
AB23A from 95% ethanol extract of *AR*	Hong Kong	In vitro, human hepatoma HepG cells resistant to vinblastine, actinomycin D, puromycin, paclitaxel, doxorubicin drugs	10 μm	vinblastine 300 nM	**↓** **Cell growth rate**	[50]
					**Cell cycle arrest** ↑G2/M arrest	
					**Chemosensitizer** **Combination index < 0.8** **↑** **C** **ellular accumulation of doxorubicin** **↓** **Pgp activity** **↓** **Rh-123 transport**	
AB23A from 95% ethanol extract of *AR*	Hong Kong	In vitro, human leukemia K562 cells resistant to vinblastine drug	10 μm	vinblastine 300 nM	**↓** **Cell growth rate**	[50]
					**Chemosensitizer** **↑** **C** **ellular accumulation of vinblastine** **→** **Pgp expression** **↓** **Rh-123 transport**	
AF24A purchased from Science and Technology Co., Ltd. (Tianjin, China)	Tianjin, China	In vitro, human colorectal adenocarcinoma Caco-2 monolayers overexpressing Pgp	10 μm	verapamil 10 μm	**↓** **Efflux ratio of digoxin**	[51]
AF24A purchased from Science and Technology Co., Ltd. (Tianjin, China)	Tianjin, China	In vitro, human breast cancer cells MCF-7-digoxin resistant cells	5, 10, 20 μm	verapamil 10 μm	**↓** **IC_50_ of doxorubicin**	[51]
					**Chemosensitizer** Log of combination index < 0 **↑Cellular accumulation of doxorubicin** **↑Nuclear migration of doxorubicin**	
					**Apoptosis induction** ↑Cytoplasmic TMRE fluorescence intensity ↑Mitochondrial membrane depolarization	
95% ethanol extract of *AR*	Hong Kong	In vitro, human hepatoma HepG2 cells resistant to vinblastine, actinomycin D, puromycin, paclitaxel, doxorubicin drugs	25 μg/ml	verapamil 10 μm	**↓Cell growth rate**	[49]
					**Cell cycle arrest** ↑G2/M arrest	
		Human leukemia K562 cells resistant to vinblastine, actinomycin D, puromycin, paclitaxel, doxorubicin drugs			**↑** **Cellular accumulation of doxorubicin** **↓** **Rh-123 transport** **→Pgp expression**	

AB23A, alisol B 23-acetate; AF24A, alisol F 24-acetate; *AR*, *Alismatis rhizome*; CDK, cyclin-dependent kinase; PARP, poly (ADP-ribose) polymerase; Pgp, *p*-glycoprotein; TMRE, tetramethylrhodamine, ethyl ester; Rh-123, rhodamine 123. Upward pointing arrows (↑) and downward pointing arrows (↓) indicate an increase or a decrease in a numerical value, rate, change, or degree, respectively.

## Data Availability

Not applicable.

## References

[1] Sung H., Ferlay J., Siegel R.L., Laversanne M., Soerjomataram I., Jemal A., Bray F. (2021). Global cancer statistics 2020: GLOBOCAN estimates of incidence and mortality worldwide for 36 cancers in 185 countries. CA A Cancer J. Clin..

[2] Kennedy N.J., Cellurale C., Davis R.J. (2007). A radical role for p38 MAPK in tumor initiation. Cancer Cell.

[3] LoRusso P.M. (2016). Inhibition of the PI3K/AKT/mTOR pathway in solid tumors. J. Clin. Oncol..

[4] Patnaik A., Haluska P., Tolcher A.W., Erlichman C., Papadopoulos K.P., Lensing J.L., Beeram M., Molina J.R., Rasco D.W., Arcos R.R. (2016). A first-in-human phase I study of the oral p38 MAPK inhibitor, ralimetinib (LY2228820 Dimesylate), in patients with advanced cancer. Clin. Cancer Res..

[5] Polivka J., Janku F. (2014). Molecular targets for cancer therapy in the PI3K/AKT/mTOR pathway. Pharmacol. Ther..

[6] Martínez-Limón A., Joaquin M., Caballero M., Posas F., de Nadal E. (2020). The p38 pathway: From biology to cancer therapy. Int. J. Mol. Sci..

[7] Yin S.-Y., Wei W.-C., Jian F.-Y., Yang N.-S. (2013). Therapeutic applications of herbal medicines for cancer patients. Evid. Based Complement. Altern. Med..

[8] Huang M., Lu J.-J., Huang M.-Q., Bao J.-L., Chen X.-P., Wang Y.-T. (2012). Terpenoids: Natural products for cancer therapy. Expert Opin. Investig. Drugs.

[9] Feng L., Liu T.T., Huo X.K., Tian X.G., Wang C., Lv X., Ning J., Zhao W.Y., Zhang B.J., Sun C.P. (2021). Alisma genus: Phytochemical constituents, biosynthesis, and biological activities. Phytother. Res..

[10] Park Y.-J., Kim M.-S., Kim H.-R., Kim J.-M., Hwang J.-K., Yang S.-H., Kim H.-J., Lee D.-S., Oh H., Kim Y.-C. (2014). Ethanol extract of Alismatis rhizome inhibits adipocyte differentiation of OP9 cells. Evid. Based Complement. Altern. Med..

[11] Wang P., Song T., Shi R., He M., Wang R., Lv J., Jiang M. (2020). Triterpenoids from Alisma species: Phytochemistry, structure modification, and bioactivities. Front. Chem..

[12] Petronelli A., Pannitteri G., Testa U. (2009). Triterpenoids as new promising anticancer drugs. Anti Cancer Drugs.

[13] Xu W., Li T., Huang M., Chen X.P., Lu J.J. (2013). Anti-cancer effects of triterpenoids isolated form *Alismatis rhizoma* on HepG2 cells. Acta Pharmacol. Sin..

[14] Ma Q., Han L., Bi X., Wang X., Mu Y., Guan P., Li L., Huang X. (2016). Structures and biological activities of the triterpenoids and sesquiterpenoids from Alisma orientale. Phytochemistry.

[15] Jin S. (2006). Autophagy, mitochondrial quality control, and oncogenesis. Autophagy.

[16] Kwon M.J., Kim J.N., Park J., Kim Y.T., Lee M.J., Kim B.J. (2021). Alisma canaliculatum Extract Affects AGS Gastric Cancer Cells by Inducing Apoptosis. Int. J. Med. Sci..

[17] Choi J., Ahn S.S., Lim Y., Lee Y.H., Shin S.Y. (2018). Inhibitory effect of Alisma canaliculatum ethanolic extract on NF-κB-dependent CXCR3 and CXCL10 expression in TNFα-exposed MDA-MB-231 breast cancer cells. Int. J. Mol. Sci..

[18] Lou C., Xu X., Chen Y., Zhao H. (2019). Alisol A suppresses proliferation, migration, and invasion in human breast cancer MDA-MB-231 cells. Molecules.

[19] Shi Y., Wang M., Wang P., Zhang T., Yu J., Shi L., Li M., Wang H., Zhang Q., Zhao H. (2020). Alisol A is potentially therapeutic in human breast cancer cells. Oncol. Rep..

[20] Xu F., Lu C., Wu Q., Chen J., Gu W., Du W., You M. (2018). Study on antitumor molecular mechanism of Alisols based on p53DNA. Int. J. Biol. Macromol..

[21] Law B.Y., Wang M., Ma D.-L., Al-Mousa F., Michelangeli F., Cheng S.-H., Ng M.H., To K.-F., Mok A.Y., Ko R.Y. (2010). Alisol B, a novel inhibitor of the sarcoplasmic/endoplasmic reticulum Ca^2+^ ATPase pump, induces autophagy, endoplasmic reticulum stress, and apoptosis. Mol. Cancer Ther..

[22] Zhang A., Sheng Y., Zou M. (2017). Antiproliferative activity of Alisol B in MDA-MB-231 cells is mediated by apoptosis, dysregulation of mitochondrial functions, cell cycle arrest and generation of reactive oxygen species. Biomed. Pharmacother..

[23] Chou C., Pan S., Teng C., Guh J. (2003). Pharmacological evaluation of several major ingredients of Chinese herbal medicines in human hepatoma Hep3B cells. Eur. J. Pharm. Sci..

[24] Xu Y.-H., Zhao L.-J., Li Y. (2009). Alisol B acetate induces apoptosis of SGC7901 cells via mitochondrial and phosphatidylinositol 3-kinases/Akt signaling pathways. World J. Gastroenterol. WJG.

[25] Huang Y.-T., Huang D.-M., Chueh S.-C., Teng C.-M., Guh J.-H. (2006). Alisol B acetate, a triterpene from Alismatis rhizoma, induces Bax nuclear translocation and apoptosis in human hormone-resistant prostate cancer PC-3 cells. Cancer Lett..

[26] Kwon M.J., Kim J.N., Lee M.J., Kim W.K., Nam J.H., Kim B.J. (2021). Apoptotic effects of alisol B 23-acetate on gastric cancer cells. Mol. Med. Rep..

[27] Wang J., Li H., Wang X., Shen T., Wang S., Ren D. (2018). Alisol B-23-acetate, a tetracyclic triterpenoid isolated from Alisma orientale, induces apoptosis in human lung cancer cells via the mitochondrial pathway. Biochem. Biophys. Res. Commun..

[28] Liu Y., Xia X.C., Meng L.Y., Wang Y., Li Y.M. (2019). Alisol B 23-acetate inhibits the viability and induces apoptosis of non-small cell lung cancer cells via PI3K/AKT/mTOR signal pathway. Mol. Med. Rep..

[29] Xia J., Luo Q., Huang S., Jiang F., Wang L., Wang G., Xie J., Liu J., Xu Y. (2019). Alisol B 23-acetate-induced HepG2 hepatoma cell death through mTOR signaling-initiated G1 cell cycle arrest and apoptosis: A quantitative proteomic study. Chin. J. Cancer Res..

[30] Li L., Cheng J., Zhu D., Shi X., Wei Y., Chen S., Wang Z., Yuan D. (2020). The effects of Alisol B 23-acetate in hepatocellular carcinoma via inducing cell apoptosis and inhibiting cell migration and invasion. Gen. Physiol. Biophys..

[31] Xu W., Li T., Qiu J.F., Wu S.S., Huang M.Q., Lin L.G., Zhang Q.W., Chen X.P., Lu J.J. (2015). Anti-proliferative activities of terpenoids isolated from Alisma orientalis and their structure-activity relationships. Anti Cancer Agents Med. Chem..

[32] Donaldson K.L., Goolsby G., Kiener P.A., Wahl A.F. (1994). Activation of p34cdc2 coincident with taxol-induced apoptosis. Cell Growth Differ..

[33] Zhang L.-L., Xu Y.-L., Tang Z.-H., Xu X.-H., Chen X., Li T., Ding C.-Y., Huang M.-Q., Chen X.-P., Wang Y.-T. (2016). Effects of alisol B 23-acetate on ovarian cancer cells: G1 phase cell cycle arrest, apoptosis, migration and invasion inhibition. Phytomedicine.

[34] Wu R., Li X.-Y., Wang W.-H., Cai F.-F., Chen X.-L., Yang M.-D., Pan Q.-S., Chen Q.-L., Zhou R.-Y., Su S.-B. (2019). Network pharmacology-based study on the mechanism of Bushen-Jianpi decoction in liver cancer treatment. Evid. Based Complement. Altern. Med..

[35] Lee S., Kho Y., Min B., Kim J., Na M., Kang S., Maeng H., Bae K. (2001). Cytotoxic triterpenoides from Alismatis rhizoma. Arch. Pharmacal Res..

[36] Shao B., Wang S., Zhou J., Ke L., Rao P. (2011). A novel lectin from fresh rhizome of Alisma orientale (Sam.) Juzep. Process Biochem..

[37] Chen H.-W., Hsu M.-J., Chien C.-T., Huang H.-C. (2001). Effect of alisol B acetate, a plant triterpene, on apoptosis in vascular smooth muscle cells and lymphocytes. Eur. J. Pharmacol..

[38] Zhao Y., Li E.T., Wang M. (2017). Alisol B 23-acetate induces autophagic-dependent apoptosis in human colon cancer cells via ROS generation and JNK activation. Oncotarget.

[39] Valastyan S., Weinberg R.A. (2011). Tumor metastasis: Molecular insights and evolving paradigms. Cell.

[40] Fares J., Fares M.Y., Khachfe H.H., Salhab H.A., Fares Y. (2020). Molecular principles of metastasis: A hallmark of cancer revisited. Signal Transduct. Target. Ther..

[41] Ma B., Xiang Y., Li T. (2003). Inhibitory effect of Alisma orientalis on spontaneous metastasis of Lewis lung carcinoma and its mechanism. Chin. Tradit. Drug.

[42] Pires B.R., Mencalha A.L., Ferreira G.M., de Souza W.F., Morgado-Díaz J.A., Maia A.M., Corrêa S., Abdelhay E.S. (2017). NF-kappaB is involved in the regulation of EMT genes in breast cancer cells. PLoS ONE.

[43] D’Alterio C., Scala S., Sozzi G., Roz L., Bertolini G. (2019). Paradoxical effects of chemotherapy on tumor relapse and metastasis promotion. Semin. Cancer Biol..

[44] Thomas H., Coley H.M. (2003). Overcoming multidrug resistance in cancer: An update on the clinical strategy of inhibiting p-glycoprotein. Cancer Control.

[45] Katayama K., Noguchi K., Sugimoto Y. (2014). Regulations of P-glycoprotein/ABCB1/MDR1 in human cancer cells. New J. Sci..

[46] Uramoto H., Tanaka F. (2014). Recurrence after surgery in patients with NSCLC. Transl. Lung Cancer Res..

[47] Castells M., Thibault B., Delord J.-P., Couderc B. (2012). Implication of tumor microenvironment in chemoresistance: Tumor-associated stromal cells protect tumor cells from cell death. Int. J. Mol. Sci..

[48] O’Connor D., Sibson K., Caswell M., Connor P., Cummins M., Mitchell C., Motwani J., Taj M., Vora A., Wynn R. (2011). Early UK experience in the use of clofarabine in the treatment of relapsed and refractory paediatric acute lymphoblastic leukaemia. Br. J. Haematol..

[49] Fong W.-F., Wang C., Zhu G.-Y., Leung C.-H., Yang M.-S., Cheung H.-Y. (2007). Reversal of multidrug resistance in cancer cells by Rhizoma Alismatis extract. Phytomedicine.

[50] Wang C., Zhang J.-X., Shen X.-L., Wan C.-K., Tse A.K.-W., Fong W.-F. (2004). Reversal of P-glycoprotein-mediated multidrug resistance by Alisol B 23-acetate. Biochem. Pharmacol..

[51] Pan G., Li T., Zeng Q., Wang X., Zhu Y. (2016). Alisol F 24 acetate enhances chemosensitivity and apoptosis of MCF-7/DOX Cells by inhibiting P-glycoprotein-mediated drug efflux. Molecules.

[52] Cuenda A., Rousseau S. (2007). p38 MAP-kinases pathway regulation, function and role in human diseases. Biochim. Biophys. Acta Mol. Cell Res..

[53] Pan S.-T., Qin Y., Zhou Z.-W., He Z.-X., Zhang X., Yang T., Yang Y.-X., Wang D., Qiu J.-X., Zhou S.-F. (2015). Plumbagin induces G2/M arrest, apoptosis, and autophagy via p38 MAPK-and PI3K/Akt/mTOR-mediated pathways in human tongue squamous cell carcinoma cells. Drug Des. Dev. Ther..

[54] Xu Y., Li N., Xiang R., Sun P. (2014). Emerging roles of the p38 MAPK and PI3K/AKT/mTOR pathways in oncogene-induced senescence. Trends Biochem. Sci..

[55] Song M., Bode A.M., Dong Z., Lee M.-H. (2019). AKT as a therapeutic target for cancer. Cancer Res..

[56] DiDonato J.A., Mercurio F., Karin M. (2012). NF-κB and the link between inflammation and cancer. Immunol. Rev..

[57] Fulda S., Debatin K.M. (2006). Extrinsic versus intrinsic apoptosis pathways in anticancer chemotherapy. Oncogene.

[58] Dong C., Wu J., Chen Y., Nie J., Chen C. (2021). Activation of PI3K/AKT/mTOR Pathway Causes Drug Resistance in Breast Cancer. Front. Pharmacol..

[59] Kim E., Viatour P. (2020). Hepatocellular carcinoma: Old friends and new tricks. Exp. Mol. Med..

[60] Suresh D., Srinivas A.N., Kumar D.P. (2020). Etiology of Hepatocellular Carcinoma: Special Focus on Fatty Liver Disease. Front. Oncol..

[61] Choi E., Jang E., Lee J.-H. (2019). Pharmacological activities of Alisma orientale against nonalcoholic fatty liver disease and metabolic syndrome: Literature review. Evid. Based Complement. Altern. Med..

